# Knockdown of Akt Sensitizes Osteosarcoma Cells to Apoptosis Induced by Cisplatin Treatment

**DOI:** 10.3390/ijms12052994

**Published:** 2011-05-10

**Authors:** Guoyou Zhang, Ming Li, Xiaodong Zhu, Yushu Bai, Changwei Yang

**Affiliations:** Department of Orthopaedics, Changhai Hospital, Second Military Medical University, Shanghai 200433, China; E-Mails: Zhangguoyou7@gmail.com (G.Z.); zhgych@yahoo.com.cn (X.Z.); baiyushu@21cn.com (Y.B.); changwei_y@yahoo.com.cn (W.Y.)

**Keywords:** osteosarcoma, Akt, PUMA, apoptosis, cisplatin, chemotherapy

## Abstract

Akt plays an important role in the inhibition of apoptosis induced by chemotherapy and other stimuli. We therefore investigated if knockdown of Akt2 promoted drug-induced apoptosis in cultured osteosarcoma cells *in vitro*. SAOS-2 cells were transfected with Akt2 siRNA. The sensitivity of the transformed cell line to the chemotherapeutic drug cisplatin was assessed. Reduced expression of Akt2 did not directly inhibit the growth rate of the transfected cells; however, it significantly increased their sensitivity to cisplatin. Knockdown of Akt2, together with cisplatin treatment, promoted the expression of p53 up-regulated modulator of apoptosis (PUMA). It is possible that the augmentation of cisplatin cytotoxicity may be mediated by PUMA activation. The results of this study suggest that knockdown of Akt2 expression may have therapeutic applications in enhancing the efficacy of chemotherapy in patients with osteosarcoma.

## 1. Introduction

Primary osteosarcoma is the most common bone tumor, and occurs predominantly in adolescents and young adults [1]. Even after the introduction of aggressive chemotherapy and wide excision of tumors, 30–50% of patients with initially localized disease subsequently develop recurrence, with subsequently poor clinical outcomes. Moreover, 20–30% of newly diagnosed cases present with metastatic disease [2,3]. The identification of the effector molecules and/or signal transduction pathways responsible for regulating carcinogenesis and malignant development is therefore crucial for understanding and isolating potential molecular targets that could be used to disrupt the tumor machinery, whilst protecting the integrity and function of normal tissue.

Akt is a serine/threonine kinase that plays a central role in tumorigenesis. Among the members of Akt family, Akt2 is associated with the development of human cancers. Recent reports have demonstrated that the PI3K/Akt pathway is a potent survival signal that may mediate resistance to the apoptotic effects of chemotherapy and radiation therapy in a variety of cancer types [4–6].

p53 up-regulated modulator of apoptosis (PUMA) is an essential mediator of cell death and plays a key functional role in the process of p53-mediated apoptosis [7,8]. PUMA activation was recently reported to be a mechanism for the augmentation of cisplatin cytotoxicity in QBC939 cells [9]. Alison *et al.* [10] recently found that Akt2 inhibition enhanced the inhibitory effects of PUMA on melanoma cell survival *in vitro* and on melanoma tumor growth *in vivo*.

In this study, we investigated the hypothesis that knockdown of Akt2 plays an important role in osteosarcoma cell chemosensitivity to cisplatin-induced apoptosis through upregulation of PUMA.

## 2. Materials and Methods

### 2.1. Cell Culture

The human osteosarcoma cell line SAOS-2 was obtained from the American Type Culture Collection (Manassas, VA, USA) and was maintained in Dulbecco’s Modified Eagle’s Medium (DMEM) supplemented with 10% fetal bovine serum, penicillin (100 U/mL) and streptomycin (100 U/mL) at 37 °C in an atmosphere of 5% CO_2_.

### 2.2. Immunohistochemistry

SAOS-2 cells were plated at a density of 5 × 10^4^/mm^2^ in culture dishes for 24 h. After incubation, dishes were washed twice with cold saline. Subsequently, the cells in the dishes were fixed in 0.3 mol/L NaCl in 70% ethanol for 30 min. The fixed cells were lysed in 0.12 N HCl in 70% ethanol for 10 min and washed with PBS for 5 min three times. The cells were incubated with 1% normal horse serum in PBS for 30 min at room temperature. After washes with PBS for 5 min, Anti-Akt1 (B-1)(1:100, Santa Cruz), Anti-Akt2 (1:100, Cell Signaling Technology, Inc., Beverly, MA, USA) and Anti-Akt3 (M-14) (1:100, Cell Signaling Technology) was used as the primary antibody, followed by the rabbit ABC staining system. Protein-positive cells were counted by light microscopy at ×200 magnification and expressed as the number of positive cells per 1000 cancer cells.

### 2.3. Small Interference (siRNA) Transfection

The following siRNA sequences were utilized in this study: Akt2-siRNA (sense 5′-UGCCCUUCUACAACCAGGAdTdT-3′), Akt-3m-siRNA (control siRNA for transfection) (sense 5′-UGCCGUUCUUCAACGAGGAdTdT-3′) [11]. The siRNA oligonucleotides, together with the corresponding antisense oligonucleotides, were synthesized by Dharmacon (Lafayette, CO, USA). The control siRNA was from Ambion (Austin, TX, USA). siRNA transfection was performed using Lipofectamine 2000, according to the manufacturer's instructions, and following procedures described previously [12]. Pilot experiments were performed to optimize the amount and time of maximal protein knockdown. Cells were treated with cisplatin following siRNA transfection, during the period of maximal protein knockdown.

### 2.4. Cell Proliferation and Cytotoxicity Assay

The effects of Akt2 silencing on growth of SAOS-2 cells were assessed by culturing control cells and cells transfected with Akt2-siRNA in the presence of 1 μg/mL cisplatin for 48 h. Cells from these precultures were seeded at 3 × 10^3^ cells/well into 96-well plates and allowed to attach for 12 h. Growth was then measured by determining the numbers of living cells using the MTT method. The results are expressed as percentage of viable cells at the start of the experiment.

For cytotoxicity assays, 5 × 10^3^ cells/well were plated in 96-well plates and incubated overnight. The medium was then replaced with fresh medium containing different concentrations of cisplatin (Eli Lilly, Geneva, Switzerland) and incubation was continued for 48 h. Cisplatin (1 μg/mL) was present throughout the experiment. Cell viability was determined using the MTT method. Percent survival was defined as 100 × (*T* − *T*_0_)/(*C* − *T*_0_) when (*T* − *T*_0_) = 0. When the *T* value was less than *T*_0_, cell killing had occurred and the cytotoxic activity was determined as (*T* − *T*_0_)/*T*_0_ expressed as a percentage. *T* is the optical density (OD)_540_ value at the time-point in question, and *T*_0_ is the OD_540_ value at the moment of drug addition. C indicates the OD_540_ value of the untreated control group at the time-point in question. IC_50_ values were calculated from three independent experiments.

### 2.5. Apoptosis Detection by Enzyme-Linked Immunosorbent Assay (ELISA)

This assay was performed using the cell death detection ELISAPLUS kit (Roche Applied Sciences, Indianapolis, IN, USA), according to the manufacturer’s instructions. Cells of both transfected and untransfected cells were plated on 6-well plates and treated with different indicated concentrations of cisplatin, after which both adherent and floating (apoptotic) populations were harvested. Cells were lysed in NP-40 lysis buffer and nucleosomes in the supernatant were detected photometrically using an ELISA plate Reader (SpectraMax 190, Molecular Devices Ca). The readings were expressed as degree of apoptosis relative to the untreated control, which was scored as 1.

### 2.6. Analysis of Apoptosis by DAPI Staining

For apoptosis analysis, both adherent and non-adherent cells were harvested after cisplatin treatment and their cell morphologies were examined by microscopy. DNA damage characteristic of apoptosis was identified by staining with 4′,6-diamidino-2-phenylindole (DAPI). Briefly, cells were harvested, washed, and fixed in 3.7% formaldehyde at room temperature for 15 min. After treatment with RNase A, samples were stained with 1 μg/mL of DAPI (Sigma USA) in phosphate-buffered saline (PBS) for 15 min at room temperature, rinsed in PBS, and analyzed using a Leica DM RXE fluorescence microscope (Leica, Wetzlar, Germany). Apoptotic cells were defined by the condensation of nuclear chromatin, fragmentation, or margination to the nuclear membrane.

### 2.7. Western Blotting

Cells were lysed in 250 mM NaCl, 50 mM Hepes (pH 7), 0.1% Nonidet P-40, and 1 μM proteinase inhibitor cocktail (Roche, Basel, Switzerland). Protein concentration was determined using the BCA protein assay (Pierce Chemical Company, Rockford, IL, USA). Equal amounts of cellular protein (20 μg/sample) were electrophoresed on 12% sodium dodecyl sulfate-polyacrylamide gels and transferred to nitrocellulose membranes (Amersham Pharmacia Biotech, Zurich, Switzerland). Depending on the experiment, the membranes were first incubated with polyclonal rabbit antibody to phospho-AKT (serine-473) (Biosource International Inc., Camarillo, CA, USA), polyclonal antibody to AKT2 (Cell Signaling Technology, Inc., Beverly, MA, USA), polyclonal antibody to PUMA (Santa Cruz Biotechnology, Santa Cruz, CA, USA), or polyclonal antibody to actin (Santa Cruz Biotechnology, Inc.). For signal detection, the blots were then incubated with peroxidase-coupled goat anti-rabbit immunoglobulin (Amersham Pharmacia Biotech). Enhanced chemiluminescence (ECL, Amersham Pharmacia Biotech) reagents were used to detect the signals, according to the manufacturer’s instructions.

### 2.8. Wt-P53 Assay

A commercial ELISA kit (Calbiochem) was used to analyze the cellular level of wt-P53. Briefly, an anti-wt-P53 monoclonal antibody (Merck-Calbiochem) was precoated onto the 96-well microtiter plate. Cells (5 × 10^5^) of transfected and untransfected treated with different indicated concentrations of Cisplatin for 48 h was then added to the well. The plate was incubated at room temperature for 2 h and the wash was repeated. A substrate solution was then added to all wells and incubated for 30 min. At this point, a stop solution was added to all wells. Color development and intensity of the color were measured using an ELISA plate reader according to the manufacturer’s instructions. A standard curve was prepared, plotting the absorbance *versus* the concentration of the wt-P53 expressed as picogram per milliliter in the original samples.

### 2.9. Statistical Analysis

Data are presented as mean ±SD. *P* values were calculated using Student’s *t* tests or two-way analysis of variance (ANOVA) using SPSS10.0 software. *P* < 0.05 was considered to be statistically significant.

## 3. Results

### 3.1. Akt 1,2,3 Expression in SAOS-2 Cells

**Figure 1.**
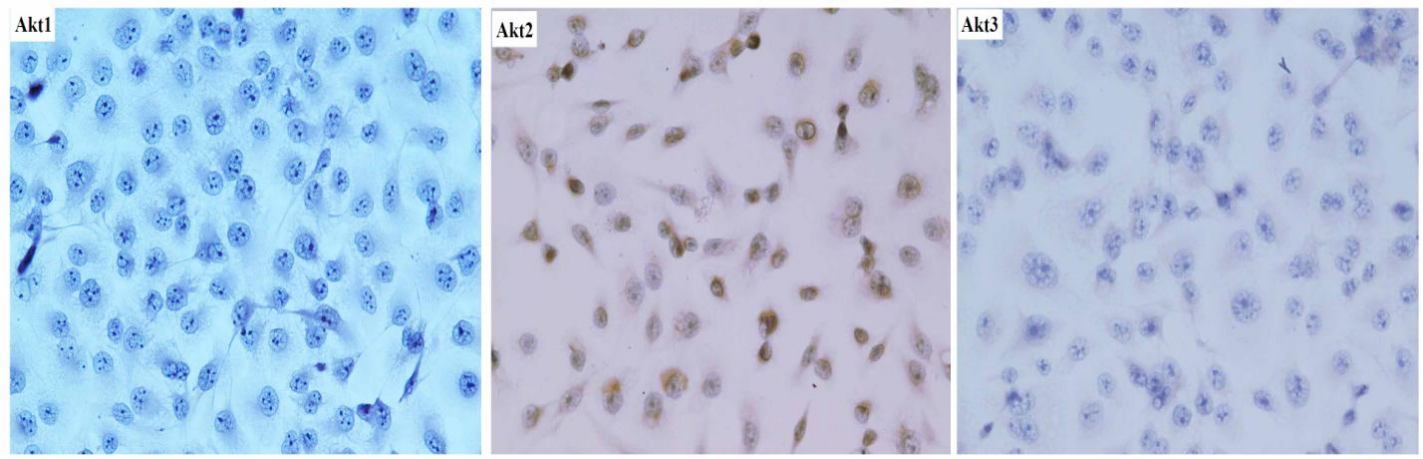
Immunohistrology analysis of levels of Slug protein in Akt 1, Akt2 and Akt3 in SAOS-2 cells. Significant Akt2 staining was seen in the cytoplasm. No obvious Akt1 and Akt3 staining was shown (BAC × 200).

Figure 1 shows the representative expression patterns of Akt1, Akt2 and Akt3 in SAOS-2 cells. Significant Akt2 staining was seen in the cytoplasm. No obvious Akt1 and Akt3 staining was shown in the SAOS-2 cells. In the present study, Akt2 was used for further study.

### 3.2. Akt2 siRNA Transfection Did not Inhibit SAOS-2 Cell Growth

**Figure 2.**
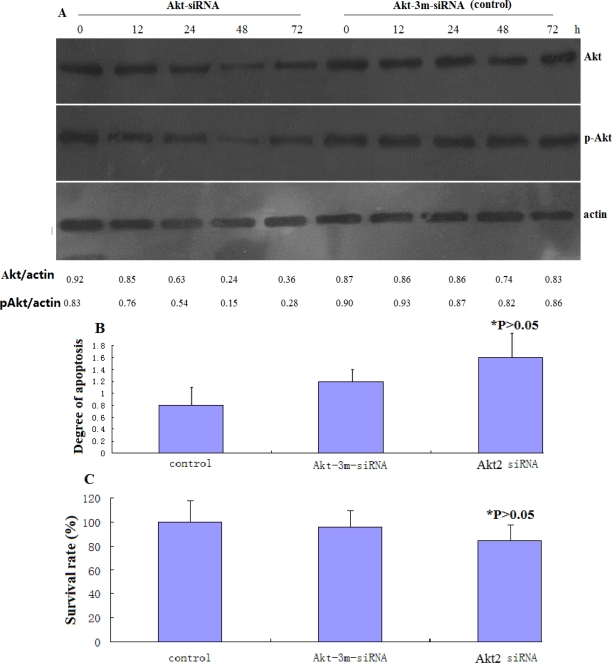
The effect of Akt2 siRNA transfection on SAOS-2 cell growth. (**A**) SAOS-2 cells were transfected with Akt2-siRNA or control Akt-3m-siRNA for 12–72 h, after which Akt2 and pAkt expression levels were analyzed by Western blotting. The expression levels of Akt2 and pAkt relative to actin are indicated. Akt2/pAkt expression was lowest in Akt2-siRNA-transfected cells after transfection for 48 h; (**B**) ELISA was used to measure the apoptosis rates in Akt2-siRNA and Akt-3m-siRNA-transfected cells after transfection for 48 h. The apoptosis rates were similar in Akt2-siRNA-transfected, Akt-3m-siRNA-transfected and control cells (* *P* > 0.05 *vs.* control); (**C**) The MTT method was used to measure the cell growth rates in Akt2-siRNA- and Akt-3m-siRNA-transfected cells after transfection for 48 h. The growth rates were similar in Akt2-siRNA- and Akt-3m-siRNA-transfected and control cells (* *P* > 0.05 *vs*. control).

SAOS-2 cells were transfected with Akt2-siRNA orAkt-3m-siRNA for 72 h. Figure 2A shows the results of Western blotting and the relative expression levels of Akt2/pAkt at different times after transfection. Akt2 protein expression was decreased in Akt2-siRNA-transfected cells. MTT and ELISA were performed to determine the effects of Akt2 siRNA transfection on growth rate and apoptosis. Untransfected control cells, cells transfected with control Akt-3m-siRNA, and cells transfected with Akt2-siRNA exhibited similar growth and apoptosis rates (Figure 2B,C). Knockdown of Akt2 thus had no apparent direct effect on host cell growth and apoptosis, as documented for other cancer cell lines [13].

### 3.3. Knockdown of Akt2 Sensitizes SAOS-2 Cells to Chemotherapeutic Agents

**Figure 3.**
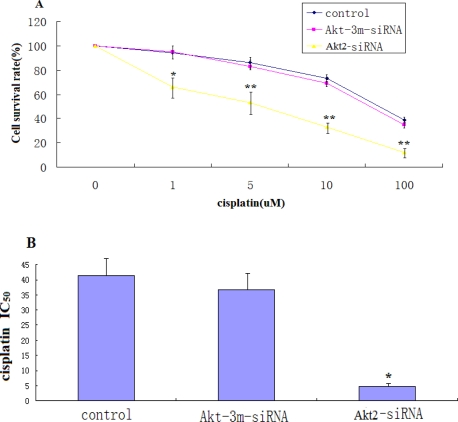
Knockdown of Akt2 sensitizes SAOS-2 cells to chemotherapeutic agents. (**A**) Cytotoxicity of cisplatin in SAOS-2 cells, SAOS-2 cells transfected with Akt2-siRNA and SAOS-2 cells transfected with Akt-3m-siRNA. Cells were treated with cisplatin at the indicated concentrations for 48 h. Percent survival was determined using the MTT assay. Each point represents the mean ± SD (error bars) from three independent experiments (** *P* < 0.05, * *P* < 0.05 *vs*. control); (**B**) IC_50_ values for cisplatin in the different cell lines. The IC_50_ values were determined after 48 h of exposure to cisplatin and were defined as the concentration causing 50% growth inhibition in treated cells, compared to that in control cells. Values are means ± SD from at least three independent experiments (* *P* < 0.05 *vs.* control).

Knockdown of Akt2 in SAOS-2 cells increased their susceptibility to cisplatin. Figure 3 shows the decrease in cell viability as a function of drug concentration following treatment with cisplatin for 48 h. Akt2-siRNA-transfected SAOS-2 cells were significantly more sensitive to cisplatin compared with both untransfected and Akt-3m-siRNA-transfected parent cells. The reduced survival of Akt2-siRNA-transfected SAOS-2 cells was most pronounced at low drug concentrations. There was no significant difference in survival between Akt-3m-siRNA-transfected cells and parent SAOS-2 cells (Figure 3A). The mean IC_50_ for cisplatin in Akt2-siRNA-transfected cells was 4.8 μM, compared to 41.4 μM for control cells (Figure 3B). This corresponds to a 9-fold increase in chemosensitivity.

### 3.4. Apoptosis

**Figure 4.**
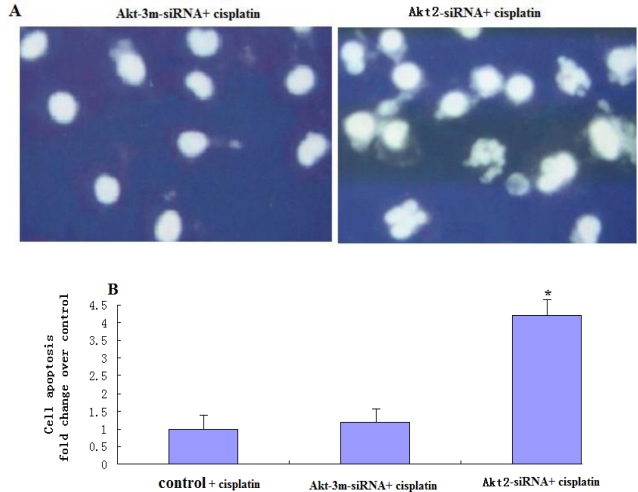
DAPI staining and ELISA for detection of apoptotic cells. (**A**) Untransfected, Akt2-siRNA-transfected, and Akt-3m-siRNA-transfected SAOS-2 cells were treated with cisplatin (1 μM) for 48 h and then fixed and stained with DAPI. Morphological changes were visualized by fluorescence microscopy. Nuclear fragmentation and apoptotic bodies were clearly apparent in Akt2-siRNA-transfected cells, while no nuclear fragmentation or apoptotic bodies were seen in Akt-3m-siRNA-transfected or untransfected SAOS-2 cells treated with cisplatin (1 μM); (**B**) Untransfected, Akt2-siRNA-transfected, and Akt-3m-siRNA-transfected SAOS-2 cells treated with cisplatin (1 μM) for 48 h. At the end of incubation cells were harvested and apoptosis assays were performed using a cell death detection ELISAPLUS kit. Data in each set represents the mean ± S.D. of three independent experiments.

Treatment of Akt2-siRNA-transfected SAOS-2 cells with low concentrations of cisplatin (1 μM) resulted in morphological changes typical of apoptosis, such as cell shrinkage, rounding and detachment of the cells from the plate, as observed by phase contrast microscopy (not shown). DAPI staining was used to visualize nucleosomal DNA damage. Nuclear fragmentation and apoptotic bodies were clearly apparent in Akt2-siRNA-transfected SAOS-2 treated with cisplatin (1 μM), while no nuclear fragmentation or apoptotic bodies were seen in Akt-3m-siRNA-transfected or untransfected SAOS-2 cells treated with cisplatin (1 μM) (Figure 4A). ELISA analysis determined that the apoptosis rate in Akt2-siRNA-transfected SAOS-2 cells treated with cisplatin (1 μM) was higher than in similarly-treated Akt-3m-siRNA-transfected or untransfected SAOS-2 cells (*P* < 0.05) (Figure 4B).

### 3.5. Effects of cisplAtin Exposure on Activation of Akt2

Following exposure to apoptotic stimuli, SAOS-2 cells may engage survival mechanisms to subvert the induction of cell death. Activation of the Akt2 signaling pathway has been observed following exposure of diverse cancer cell types to various chemotherapeutic agents. In the present study, SAOS-2 cells were treated with cisplatin (10 μM) and the pAkt levels were examined over a period of 2–6 h. Cisplatin treatment induced a rapid increase in Akt2/pAkt levels (Figure 4).

### 3.6. Knockdown of Akt2 Combined with Cisplatin Treatment Upregulates PUMA

**Figure 6.**
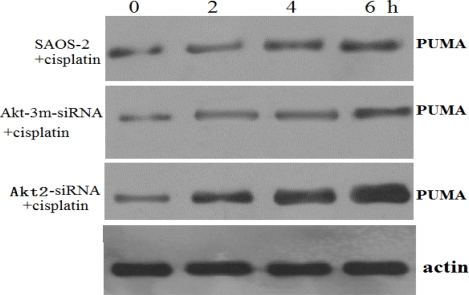
Knockdown of Akt2 combined with cisplatin treatment upregulates PUMA. Western blot analysis of PUMA in untransfected, Akt2-siRNA-transfected, and Akt-3m-siRNA-transfected SAOS-2 cells treated with cisplatin (10 μM) for 2–6 h. A rapid increase in PUMA levels occurred in Akt2-siRNA-transfected SAOS-2 treated with cisplatin.

Chemotherapeutic agents have previously been found to activate PUMA [9]. In the present study, treatment of Akt2-siRNA-transfected SAOS-2 with cisplatin (10 μM) for 2–6 h induced a rapid increase in PUMA levels (Figure 6). These findings suggest that sensitization of cells by Akt2 silencing proceeds via PUMA activation.

### 3.7. Apoptosis Induction by Akt2 Knockdown and Cisplatin Treatment Is p53 Independent

**Figure 7.**
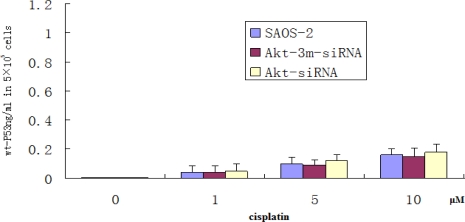
ELISA assay of wt-P53 in the supernatant medium. No detectable wt-P53 protein was shown in untransfected, Akt2-siRNA-transfected, and Akt-3m-siRNA-transfected SAOS-2 cells, respectively. Although the wt-P53 level increased the cisplatin dose dependence, it was not significant (*P* > 0.05).

To determine whether apoptosis induction by Akt2 knockdown and cisplatin treatment is p53 independent, ELISA method was used to detect wt-P53 level in SAOS-2 cells treated with Akt2 knockdown and/or cisplatin treatment (1, 5 and 10 μM). As shown in Figure 7, no detectable wt-P53 protein was shown in untransfected, Akt2-siRNA-transfected, and Akt-3m-siRNA-transfected SAOS-2 cells, respectively. Although the wt-P53 level increased the cisplatin dose dependence, it was not significant (*P* > 0.05).

## 4. Discussion

The therapeutic advantages of cisplatin, such as high efficiency, mild side effects and easy administration, mean that it is still one of the most commonly used chemotherapeutic agents. However, resistance to cisplatin often occurs, and methods of enhancing the sensitivity of cancer cells to cisplatin-induced apoptosis have therefore become an important chemotherapeutic strategy.

The PI3K/Akt survival pathway is activated by survival signals such as growth factors, cytokines, hormones and oncogenic Ras [14]. Activation of Akt favors survival via the direct regulation of apoptotic proteins, including the Bcl-2 members Bad and Bcl-xl or caspase 9 [15–18]. Akt signaling has recently been shown to mediate therapeutic resistance. The PI3K/Akt pathway is frequently overexpressed/activated in cancers, and Akt activation promotes a chemoresistant phenotype, whereas Akt inhibition sensitizes chemoresistant cells to cisplatin-induced apoptosis.

In the present study, inhibition of Akt2 had minimal effect on the basal level of apoptosis, though it reduced the threshold for the induction of apoptosis in response to chemotherapy in SAOS-2 cells. These data suggest that targeted inhibition of the Akt2 pathway may not be an adequate therapy when administered alone. However, we also examined the ability of Akt2 inhibition to sensitize SAOS-2 cells, and demonstrated increased apoptosis after combined Akt2 inhibition and cisplatin treatment, suggesting that high basal Akt2 activity is required for the efficacy of this targeted therapy. The mechanism by which Akt2 inhibition confers chemosensitivity in these cancer cells is unclear, but it appears to involve the regulation of transcription factors and proapoptotic proteins, such as PUMA. Recent evidence suggests that upregulation of PUMA is an important mechanism in cisplatin-induced apoptosis [19], and Akt contributes to chemoresistance by attenuating p53-mediated PUMA upregulation and phosphorylation of p53, which are essential but independent determinants of cisplatin sensitivity [20]. The results of this study showed that SAOS-2 cells responded to chemotherapy exposure with the induction of an Akt2-dependent survival pathway that may involve Akt2-mediated transcriptional inhibition of the proapoptotic PUMA gene. Akt2 inhibition enhanced the proapoptotic effect of chemotherapy via upregulating the induction of PUMA. To determine whether apoptosis induction by Akt2 knockdown and cisplatin treatment is p53 independent, ELISA method was used to detect wt-P53 level in SAOS-2 cells treated with Akt2 knockdown and/or cisplatin treatment. The results showed that although the wt-P53 level increased in a cisplatin dose dependent manner, it was not significant. It was also proven that apoptosis induction by Akt2 knockdown and cisplatin treatment is p53 independent.

Chemotherapy appears to wield a double-edged sword; although it is able to induce apoptosis in cancer cells, it is also able to stimulate the tumor cells’ native survival mechanisms. In the present study, SAOS-2 cells on the one hand responded to chemotherapy exposure by induction of an Akt2-dependent survival pathway, while on the other hand, chemotherapy exposure induced proapoptotic PUMA signaling. The benefits of combining Akt2 inhibition with standard chemotherapy thus appear to be twofold; disruption of the Akt2 survival pathway in cancer cells that harbor constitutively active Akt may induce an apoptotic response, while inhibition of Akt may abrogate the undesired survival response seen when tumor cells are exposed to chemotherapy. The chemoresistance of SAOS-2 cells may be both a manifestation of their inherent properties, but may also reflect their ability to respond to an apoptotic stimulus with the induction of a robust cell survival response that circumvents the proapoptotic effect of chemotherapy.

## 5. Conclusions

Akt2 blocks PUMA upregulation induced by cisplatin in SAOS-2 cells, thereby conferring resistance to cisplatin-induced apoptosis. Inhibition of this survival response represents an attractive method for chemosensitization of this lethal malignancy, and the results of the current study confirm the Akt/PUMA pathway as an appropriate therapeutic target.

## Figures and Tables

**Figure 5.**
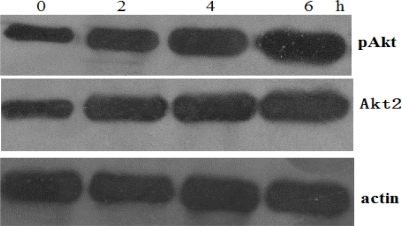
Determination of total Akt2 and phospho-Akt (pSer473) by Western blotting following treatment of SAOS-2 cells with cisplatin. SAOS-2 cells were treated with cisplatin (10 μM) and the Akt2/pAkt levels were examined over a period of 2–6 h. Cisplatin treatment induced a rapid increase in Akt2/pAkt levels.
